# Increased Levels of Phosphorylated ERK Induce CTGF Expression in Autophagy-Deficient Mouse Hepatocytes

**DOI:** 10.3390/cells11172704

**Published:** 2022-08-30

**Authors:** Hye-Young Seo, So-Hee Lee, Eugene Han, Jae Seok Hwang, Mi Kyung Kim, Byoung Kuk Jang

**Affiliations:** 1 Department of Internal Medicine, Keimyung University School of Medicine, Daegu 42601, Korea; 2 Institute for Medical Science, Keimyung University School of Medicine, Daegu 42601, Korea

**Keywords:** autophagy, CTGF, ERK, ATG7, hepatocyte

## Abstract

Autophagy performs essential cell functions in the liver through an intracellular lysosomal degradation process. Several studies have reported that autophagy deficiency can lead to liver injury, including hepatic fibrosis; however, the mechanisms underlying the relationship between autophagy deficiency and liver pathology are unclear. In this study, we examined the expression levels of fibrosis-associated genes in hepatocyte-specific ATG7-deficient mice. The expression levels of the connective tissue growth factor (CTGF) and phosphorylated ERK (phospho-ERK) proteins were increased significantly in primary hepatocytes isolated from hepatocyte-specific ATG7-deficient mice compared to those isolated from control mice. In addition, the inhibition of autophagy in cultured mammalian hepatic AML12 and LX2 cells increased CTGF and phospho-ERK protein levels without altering CTGF mRNA expression. In addition, the autophagy deficiency-mediated enhancement of CTGF expression was attenuated when ERK was inhibited. Overall, these results suggest that the inhibition of autophagy in hepatocytes increases phospho-ERK expression, which in turn increases the expression of CTGF, a biomarker of fibrosis.

## 1. Introduction

The connective tissue growth factor (CTGF), called CCN2, is a secreted protein belonging to the CCN (CYR61/CTGF/NOV) family. The expression of CTGF was reported initially in endothelial cells and fibroblasts involved in connective tissue regeneration and wound healing, although it has since been detected in many other tissues. CTGF participates in the regulation of a variety of important cellular functions, such as cell adhesion, proliferation, and differentiation [1,2,3,4]. In addition, CTGF may promote the progression of fibrosis directly, or may act as a downstream mediator of transforming growth factor β (TGF-β) [5,6]. The increased expression of CTGF has been reported in the fibrotic liver, kidney, and lung, as well as in systemic sclerosis. Therefore, CTGF is used as a biomarker of fibrosis [7,8].

As a major intracellular pathway for the catabolic degradation of cytoplasmic proteins and organelles, autophagy occurs in all types of cells, although its exact process depends on the specific organ and cell type. During autophagy, intracellular components are sequestered by the autophagosome and degraded upon fusion with the lysosome [9]. Autophagy-associated genes (ATGs) control the autophagic process; in particular, ATG7 is an essential mediator of autophagosome formation [10]. Autopahgy contributes to liver homeostasis via roles in energy balance and the quality control of the cytoplasm, which are mediated by the removal of misfolded proteins, damaged organelles, and lipid droplets [11,12]. Several groups have reported that the dysregulation or deficiency of autophagy in the liver induces hepatic diseases [13,14]. However, the cellular mechanisms underlying the pathological effects of hepatic autophagy deficiency are unclear. Here, we found that the expression levels of CTGF and phospho-ERK (p-ERK) were increased significantly in hepatocyte-specific ATG7-deficient mice

## 2. Materials and Methods

### 2.1. Generation of ATG7^flox/flox^-Albumin-Cre Mice

ATG7 floxed mice (ATG7^f/f^) mice were obtained from Dr. Myung-Shik Lee (Yonsei University) with the permission from Dr. Masaaki Komatsu (Tokyo Metropolitan Institute of Medical Science) [10,15]. ATG7 floxed mice (ATG7^f/f^) and albumin-Cre mice were bred to generate hepatocyte-specific ATG7 knockout mice (ATG7^f/f^-Cre^+^). ATG7^f/f^-Cre^+^ mice were crossed with ATG7^f/f^ or ATG7^f/+^ mice to breed ATG7^f/f^, ATG7^f/f^-Cre^+^, ATG7^f/+^, ATG7^f/+^-Cre^+^, ATG7^+/+^ and ATG7^+/+^-Cre^+^ mice were generated. ATG7^f/f^-Cre^+^ mice were born healthy and fertile for 1 year with no noticeable pathological phenotype. All animal experiments were approved by the Institutional Animal Care and Use Committee of Keimyung University (KM-2019-14R3) and were performed in accordance with the institutional guidelines for animal research. Mice were genotyped using the following primers to differentiate between heterozygotes and homozygotes: ATG7 flox forward: 5′-CATCTTGTAGCACC GCTGACCTGG-3′, ATG7 flox reverse: 5′-CCACTGGCCCATCAGTGAGCA TG-3′, ATG7 flox reverse: 5′-GCGGATCCTCGTATA ATGTATGCTATACGAAGTTAT-3′; Albumin-Cre forward: 5′-ACCTGAGATGTTCG CGATTATCT-3′, Albumin-Cre reverse: 5′-ACCGTCAGTACGTGAGATATCTT-3′.

### 2.2. Chemicals and Antibodies

BFM (B1793), CQ (C6628), MG132 (M7449) and U0126 (662005) were purchased from Sigma-Aldrich. Recombinant human TGF-β (5 ng/mL) was purchased from R&D Systems, and PD98059 (9900) was purchased from Cell Signaling Technology. The following antibodies were used: Anti-CTGF (Santa Cruz Biotechnology, Dallas, TX, USA, SC101586, Cell Signaling Technology, Danvers, MA, USA, SC-365970), p62/SQSTM1 (Abcam, Cambridge, UK, ab56416), collagen (Abcam, ab34710), LC3 (Cell Signaling Technology, CS2775), ATG7 (Cell Signaling Technology, CS2631), phospho-ERK (Th202/Tyr204) (Cell Signaling Technology, CS4370), ERK (Cell Signaling Technology, SC9102), GAPDH (Cell Signaling Technology, CS2118), phospho-SMAD3(Ser423/425) (Cell Signaling Technology, CS9520), and SMAD3 (Cell Signaling Technology, CS9513).

### 2.3. Immunohistochemical Analysis

Liver tissue was fixed in 10% formalin and then paraffin-embedded. Sectioning, hematoxylin and eosin (H&E) and Sirius red staining were performed according to standard protocols. Immunohistochemical staining was used for blocking and antibody dilution using antibody Diluent (Thermo Scientific, 003128, Waltham, MA, USA). Antibodies were primary antibodies against CTGF (1:200) and collagen (1:500) followed by horseradish peroxidase-conjugated anti-rabbit (Dako, K4003, Glostrup, Denmark) IgG secondary antibodies was used. Tissues were imaged using a Carl Zeiss microscope and all data were normalized to ATG7^f/f^ mice. ImageJ software (version 1.52a) (NIH, Bethesda, MD, USA) was used for quantification of immunostaining.

### 2.4. Cell Culture 

Mouse hepatocyte cell line (AML12, CRL-2254), rat kidney fibroblast cell line (NRK49F, CRL-1570) and mouse mesangial cell line (SV40 MES 13, CRL-1927) were obtained from the American Type Culture Collection. The human hepatic stellate cell line (LX2) was gifted by Dr. Won Il Jeong (KAIST, Daejeon, Korea). AML12 cells were cultured in DMEM/F12 (GIBCO-BRL) containing 10% fetal bovine serum (FBS), dexamethasone (40 ng/mL; Sigma-Aldrich, St. Louis, MI, USA), ITS (GIBCO-BRL) and antibiotics (GIBCO-BRL). LX2 cells were DMEM (GIBCO-BRL) containing 10% FBS and antibiotics. NRK49F and SV40 MES 13 cells were DMEM containing 5% FBS and antibiotics. All cells were treated with or without chemicals in medium containing 0.5% FBS. 

### 2.5. Isolation of the Primary Hepatocytes 

Male 9-13 week-old ATG7^f/f^ and ATG7^f/f^-Cre^+^ mouse hepatocytes were perfused with EGTA solution (pH 7.2) at a rate of 5 mL/min to remove all blood. Hepatocytes were isolated by perfusion of the following collagenase solution (pH 7.2) [collagenase type I (Worthington Biochemical Corp., LS004197, Lakewood, NJ, USA)] at a rate of 5 mL/min for 10 minutes. The liver was then shaken at 37 °C for 10 min, filtered through a 70 µm nylon mesh, and then centrifuged at 43× *g* at 4 °C for 5 min. The pelleted hepatocytes were resuspended in William’s Medium E (Sigma-Aldrich, W4128) and cultured in type I collagen-coated dishes (BD BIOCOA, 354401). The viability of hepatocytes was always greater than 90%. After incubation for 1-2 h, the medium was replaced with medium 199 (Sigma-Aldrich, M4530).

### 2.6. Depletion of SQSTM1/p62 Using siRNA 

The designed SQSTM1/p62-*siRNA* (*si-p62*)(SC35233) and a control-*siRNA* (*siCon*)(SC37007) were purchased from Santa Cruz Biotechnology. Cells were transfected with 100 nM *siRNA* using Lipofectamine^TM^ RNAiMAX (Thermo Scientific, 13778-075), cultured in 0.5% FBS medium, and harvested 48 h after transfection.

### 2.7. Western Blotting

The cells were collected and lysed in RIPA buffer (Thermo Scientific, 89900) containing protease/phosphatase inhibitors (genDEPOT, Katy, TX, USA). The cells were vortexed 3–4 times for 30 min on ice, and then centrifuged at 13,000 rpm for 10 min. The supernatants were collected, separated by SDS-PAGE and transferred to polyvinyl difluoride membrane. The membrane was incubated with primary antibody and then incubated with secondary antibody (Cell signaling). Protein bands were detected suing enhanced Clarity™ Western ECL substrate kit (Bio-Rad). Quantitated was performed using ImageJ software (version 1.52a).

### 2.8. Quantitative Real-Time RT-PCR 

Total RNA was isolated using TRIzol reagent (Invitrogen, 15596018, Waltham, MA, USA). cDNA was prepared using the RevertAid First Strand cDNA Synthesis Kit (Thermo Scientific) and real-time RT-PCR was performed using SYBR Green PCR Master Mix Kit. PCR primers were as follows: 45 cycles of 95 °C for 30 s, 60 °C for 10 s, and 72 °C for 15 s. The primer sequences were as follows: mouse ATG7 forward, 5′-TCGAAAACCCCATGCTCCTC-3′, and reverse, 5′-AGGGCCTGGATCTGTTTTGG-3′; mouse collagen: forward, 50-GCCTTGGAGGAAACTTTGCTT-30 and reverse,50-GCACGGAAACTCCAGCTGAT-30; mouse CTGF forward, 5′-CCAGACCCAACTATGATGCG-3′, and reverse, 5′-GTGTCCGGATGCACTTTTTG-3′; mouse TGF-β forward, 5′-AAATCAACGGGATCAGCCCC-3′, and reverse, 5′-GGATCCACTTCCAACCCAGG-3′; mouse PAI-1 forward, 5′-AAATCCCACACAGCCCATCA-3′, and reverse, 5′-GGACCACCTGCTGAAACACTTT-3′; and mouse GAPDH forward, 5′- ACGACCCCTTCATTGACCTC-3′, and reverse, 5′-ATGATGACCCTTTTGGCTCC-3′ (Bio-Rad, Richmond, CA, USA). GAPDH was used as an internal standard.

### 2.9. Immunofluorescence (IF) Analysis

Cells were fixed in 10% formalin for 10 min and then permeabilized with 0.1% Triton-X100 for 15 min. After incubation with CTGF (Santa Cruz Biotechnology, SC101586, Dallas, TX, USA), LC3 (Cell Signaling Technology, CS2775) and phospho-ERK (Cell Signaling Technology, CS4370) antibody and a Alexa Fluor conjugated secondary antibody (Invitrogen, A11001, A21244, Waltham, MA, USA) (Jackson immunoresearch, 711-165-152, West Grove, PA, USA), the cells were imaged using a confocal microscope (Carl Zeiss, Oberkochen, Germany).

### 2.10. Statistical Analysis

All values are expressed as the mean ± standard errors of the mean. Data were analyzed by one-way ANOVA or a two-tailed Student’s *t test* (GraphPad Prism 5.0); Differences in *p* values for which *p* < 0.05 and *p* < 0.01 were considered to be statistically significant. 

## 3. Results

### 3.1. Loss of ATG7 in the Liver Increases the Expression Levels of Fibrosis-Related Genes

**Figure 1:**
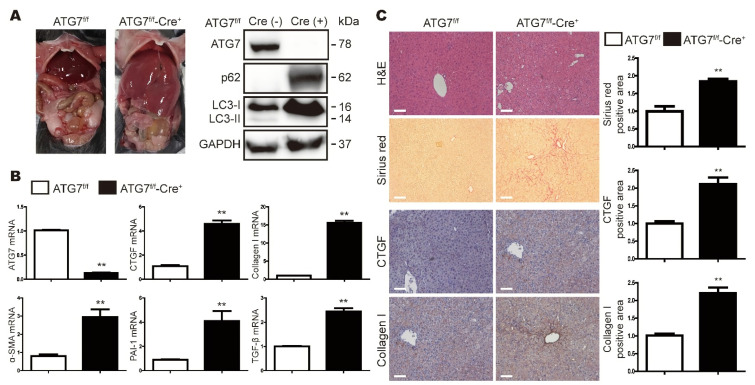
Loss of ATG7 in the mouse liver leads to fibrosis. (**A**) Gross anatomical views of the liver in representative 8-week-old male Cre-negative and Cre-positive ATG7^Flox/Flox^ (ATG7^f/f^ and ATG7^f/f^-Cre^+^) mice. Total liver lysates from ATG7^f/f^ and ATG7^f/f^-Cre^+^ mice were subjected to the Western blot analysis of ATG7, p62 and LC3. GAPDH expression was measured as a loading control. (**B**) Quantitative RT-PCR analyses of the indicated mRNAs in liver extracts from ATG7^f/f^ and ATG7^f/f^-Cre^+^ mice. The data represent the mean ± SEM (n = 8–10), and are normalized to the levels in ATG7^f/f^ mice. The data represent the mean ± SEM (n = at least 10 per group), and are normalized to the levels in ATG7^f/f^ mice. ** *p* < 0.01. (**C**) Hematoxylin and eosin (H&E), Sirius red, and immunohistochemical staining of liver tissue sections from ATG7^f/f^ and ATG7^f/f^-Cre^+^ mice. Each group contained 5–7 mice, and representative images are shown. The original magnification was ×100; the scale bars indicate 100 μm. The data in the graphs represent the mean ± SEM of five random fields per liver, and are normalized to the levels in ATG7^f/f^ mice; ** *p* < 0.01.

To examine whether autophagy deficient affects hepatic gene expression, the mRNA levels of several fibrosis-related gene were measured in total liver extracts from Cre-negative and Cre-positive ATG7^Flox/flox^ (ATG7^f/f^ and ATG7^f/f^-Cre^+^) mice. As seen in other studies involving liver-specific deletion of ATG [14,16], the liver weight (ATG7^f/f^: 1.14 ± 0.062 g; ATG7^f/f^-Cre^+^: 8.53 ± 0.58 g) was higher in ATG7^f/f^-Cre^+^ mice than in control mice (Atg7^f/f^). In ATG7^f/f^-Cre^+^ mice liver, ATG7 and LC3 II expression were not observed, and p62 expression was increased, thus confirming that autophagy did not occur (Figure 1A). Expression levels of the CTGF, collagen, α-smooth muscle actin (α-SMA), Plasminogen activator inhibitor-1 (PAI-1), and TGF-β mRNA were higher in ATG7^f/f^-Cre^+^ mice than in control mice (Figure 1B). Sirius red staining confirmed that fibrosis was more pronounced in ATG7^f/f^-Cre^+^ mice than in control mice (ATG7^f/f^). In addition, immunohistochemical staining revealed that the expression of type I collagen and CTGF were higher in ATG7^f/f^-Cre^+^ mice (Figure 1C). Taken together, these data suggest that deletion of ATG7 promotes hepatic fibrosis in mice. 

### 3.2. CTGF Expression Is Increased in ATG7-Deficient Primary Hepatocytes

**Figure 2:**
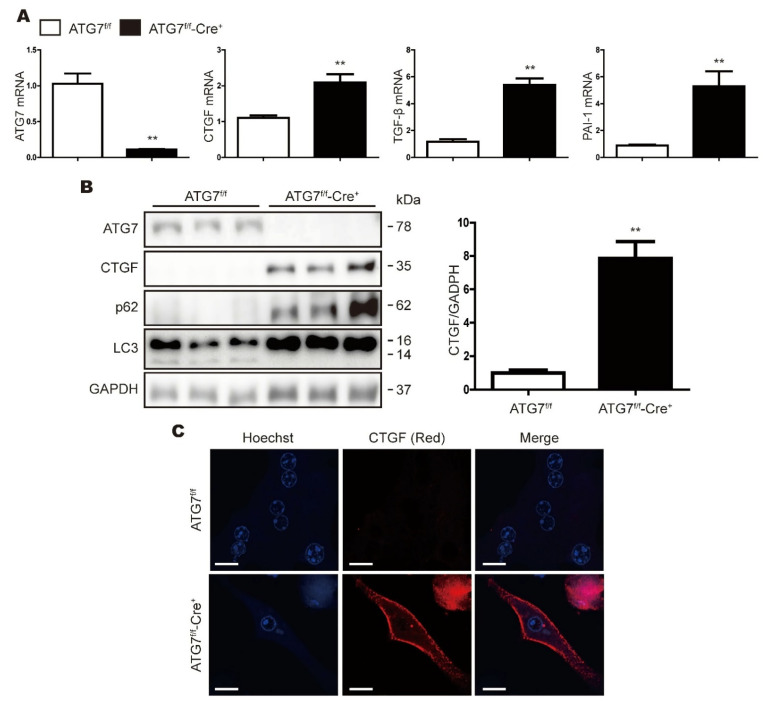
CTGF expression is increased in primary hepatocytes from Cre-positive ATG7 ^Flox/Flox^ mice. (**A**) Real-time RT PCR analysis of ATG7, CTGF, TGF-β and PAI-1 mRNA expression in primary hepatocytes from Cre-negative and Cre-positive ATG7^flox/flox^ (ATG7^f/f^ and ATG7^f/f^-Cre^+^) mice (n = at least 10 per group). The data represent the mean ± SEM, and are normalized to the levels in ATG7^f/f^ mice; ** *p* < 0.01. (**B**) Representative Western blot analysis of CTGF, SQSTM1/p62 and LC3 in ATG7^f/f^ and ATG7^f/f^-Cre^+^ mice. GAPDH expression was measured as a loading control. The data in the graph represent the mean ± SEM (n = at least 10 per group), and are normalized to the levels in ATF7^f/f^ mice; ** *p* < 0.01. (**C**) Representative IF microscopy analyses of CTGF in ATG7^f/f^ and ATG7^f/f^-Cre^+^ mice. The cell nuclei are shown in blue.

The deletion of ATG7 in mice by albumin-Cre mainly affects hepatocytes; therefore, we examined the expression levels of fibrosis markers in primary hepatocytes isolated from ATG7^f/f^ and ATG7^f/f^-Cre^+^ mice. As shown in Figure 2A, the expression levels of the mRNAs encoding CTGF, TGF-β and PAI-1 were higher in primary hepatocytes from ATG7^f/f^-Cre^+^ mice than in those from ATG7^f/f^ mice. In primary hepatocytes isolated from ATG7^f/f^-Cre^+^ mice, ATG7 was not expressed, LC3 I to LC3 II conversion did not occur, and SQSTM1/p62 expression was increased, confirming that autophagy did not occur (Figure 2B). In addition, the CTGF protein level was increased significantly following the deletion of ATG7 (Figure 2B). As is consistent with these results, immunofluorescent (IF) staining confirmed that CTGF expression was increased in ATG7^f/f^-Cre^+^ hepatocytes relative to the control hepatocytes (ATG7^f/f^) (Figure 2C). Overall, these data suggest that CTGF expression is increased significantly in autophagy-deficient hepatocytes. 

### 3.3. Inhibition of Autophagy Increases CTGF Expression in Hepatic Cell Lines

**Figure 3:**
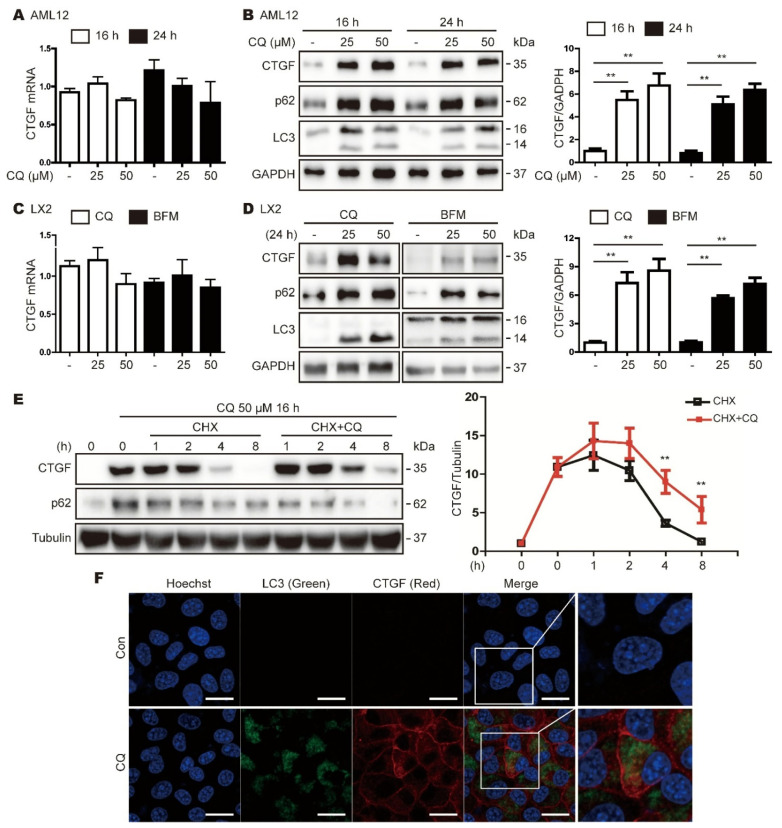
Autophagy inhibitors increase CTGF expression. (**A**,**B**) AML12 cells were treated with 25 or 50 µM CQ for 16 or 24 h (**A**) Representative real-time RT-PCR analyses of CTGF mRNA expression in AML12 cells treated with or without CQ. The data represent the mean ± SEM (n = 3). (**B**) Western blot analyses showing the effects of CQ on CTGF expression in AML12 cells. The data in the bar graph are means ± SEM of three independent measurements. ** *p* < 0.01. (**C**,**D**) LX2 cells were treated with 25 or 50 µM CQ for 24 h, or with 25 or 50 nM BFM for 24 h. (**C**) Representative real-time RT-PCR analyses of CTGF mRNA expression in LX2 cells. The data represent the mean ± SEM (N = 3). (**D**) Western blot analyses showing the effects of CQ or BFM on CTGF expression in LX2 cells. The data in the bar graph are means ± SEM of three independent measurements. ** *p* < 0.01. (**E**) Western blot analysis showing the effect of CQ on the stability of the CTGF protein in cells treated with cycloheximide (CHX) to inhibit protein synthesis. The cells were treated with or without CQ (50 µM) for up to 16 h, and protein expression was determined at 1, 2, 4 and 8 h. Tubulin expression was measured as a loading control. The data in the graph represent the mean ± SEM. (n = 4); ** *p* < 0.01. (**F**) IF staining of CTGF (red) and LC3 (green) in AML12 cells treated with or without CQ for 24 h. Con, control.

Next, we determined whether the inhibition of autophagy increases CTGF protein expression in mammalian hepatic cell lines. The treatment of mouse AML12 cells with the autophagy inhibitor chloroquine (CQ; 25 or 50 µM) increased CTGF protein expression but did not affect CTGF mRNA expression (Figure 3A,B). In addition, CTGF protein expression was increased in human LX2 hepatic stellate cells treated with CQ (25 or 50 µM) or bafilomycin A1 (BFM; 25 or 50 nM), with no corresponding change in the CTGF mRNA level (Figure 3C,D). Subsequently, we investigated whether the CQ-mediated elevation of CTGF protein levels was the result of enhanced protein stability. To this end, AML12 cells were treated with or without CQ in the presence of cycloheximide, a protein synthesis inhibitor, for various times, and CTGF protein levels were examined by Western blotting. The stability of the CTGF protein was increased significantly in the presence of CQ (Figure 3E). In addition, we used confocal fluorescence microscopy and IF staining to examine the distributions of CTGF and LC3 in AML12 cells treated with or without CQ. Consistent with the Western blotting analyses, CQ increased the levels of LC3 and CTGF, which were colocalized in the cells (Figure 3F). Taken together, these data suggest that CTGF levels are increased in hepatocytes via autophagy inhibition. 

### 3.4. ATG7 Deficiency-Induced Expression of CTGF Does Not Rely on the TGF-β Pathway

**Figure 4:**
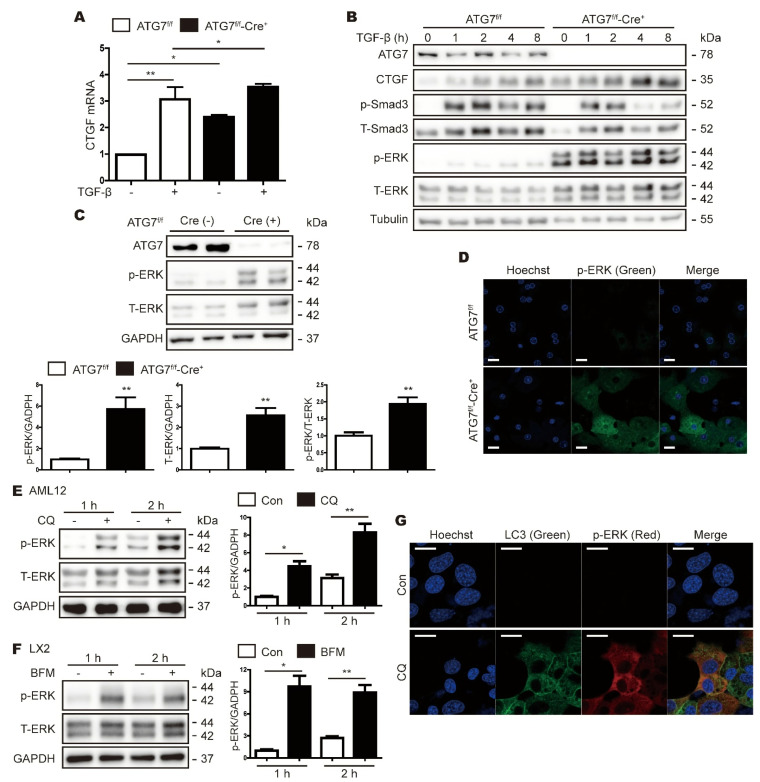
Phosphorylated ERK expression is increased in primary hepatocytes from Cre-positive ATG7^flox/flox^ mice. (**A**) Real-time RT-PCR analysis of CTGF mRNA expression in primary hepatocytes from Cre-negative and Cre-positive ATG7^flox/flox^ (ATG7^f/f^ and ATG7^f/f^-Cre^+^) mice after treatment with or without TGF-β (5 ng/mL) for 24 h. The data represent the mean ± SEM (n = 4); * *p* < 0.05, ** *p* < 0.01. (**B**) Western blot analyses of ATG7, CTGF, phospho-SMAD3 (p-SMAD3), total-SMAD3 (T-SMAD3), p-ERK, and total ERK (T-ERK) in primary hepatocytes from ATG7^f/f^ and ATG7^f/f^-Cre^+^ mice after treatment with or without TGF-β (5 ng/mL) for the indicated times. The expression of tubulin was measured as a loading control. (**C**) Representative Western blot analyses of ATG7, p-ERK, and T-ERK in primary hepatocytes from ATG7^f/f^ and ATG7^f/f^-Cre^+^ mice. GAPDH expression was measured as a loading control. The data in the graphs represent the mean ± SEM of n = 3 replicates; ** *p* < 0.01. (**D**) IF microscopy analysis of p-ERK (green) expression in primary hepatocytes from ATG7^f/f^ and ATG7^f/f^-Cre^+^ mice. The cell nuclei are visualized in blue. (**E**,**F**) Western blot analyses showing the effects of CQ (**E**) and BFM (**F**) on p-ERK levels in AML12 (**E**) and LX2 (**F**) cells, respectively. GAPDH expression was measured as a loading control. The data in the bar graphs represent the mean ± SEM of three independent measurements; * *p* < 0.05, ** *p* < 0.01. (**G**) IF microscopy analyses of p-ERK and LC3 in AML12 cells treated with or without CQ. The nuclei are visualized in blue.

In the fibrotic liver, CTGF plays a role in regulating the extracellular matrix downstream of the TGF-β signaling pathway. In order to determine whether autophagy inhibition caused by the deletion of ATG7 increases CTGF expression via the TGF-β signaling pathway, we treated primary hepatocytes from ATG7^f/f^ and ATG7^f/f^-Cre^+^ mice with or without TGF-β, and examined CTGF mRNA and CTGF protein levels via real-time RT-PCR and Western blotting, respectively. TGF-β increased CTGF mRNA expression significantly in the control hepatocytes (ATG7^f/f^) but not ATG7^f/f^-Cre^+^ hepatocytes (Figure 4A). In addition, TGF-β induced parallel increases in the levels of the CTGF and phospho-Smad3 proteins in the control hepatocytes (ATG7^f/f^). Phospho-Smad3 expression was also increased by TGF-β in ATG7^f/f^-Cre^+^ hepatocytes, although the increase was more transient than that in the control cells, and did not parallel the increases in CTGF expression (Figure 4B). In addition, the expression level of p-STAT3 was not associated with an increase in CTGF expression in ATG7^f/f^-Cre^+^ hepatocytes (Supplementary Figure S1). Overall, these data suggest that autophagy-deficient hepatocytes do not rely on TGF-β to induce CTGF expression.

### 3.5. Inhibition of Autophagy Increases ERK Expression

Previous studies have reported that CTGF is associated with the mitogen-activated protein kinase pathway [17,18,19]; therefore, we investigated changes in the expression levels of phosphorylated ERK, JNK, and p38 upon the inhibition of autophagy. In ATG7^f/f^-Cre^+^ hepatocytes, there was little expression of phospho-p38 and phospho-JNK, but the expression levels of p-ERK and total ERK were significantly higher than those in the control mice (ATG7^f/f^) (Figure 4B,C and Supplementary Figure S1). The ratio of p-ERK to total ERK was also higher in ATG7^f/f^-Cre^+^ hepatocytes than in the control hepatocytes (ATG7^f/f^), and the increase in p-ERK levels in the former was confirmed by IF staining (Figure 4D). Consistent with these findings, Western blot and IF analyses revealed that p-ERK expression was increased in AML12 and LX2 cells treated with the autophagy inhibitor CQ (Figure 4E–G). 

### 3.6. CTGF Increase in Autophagy Inhibition Is Associated with the Upregulation of Phospho-ERK

**Figure 5:**
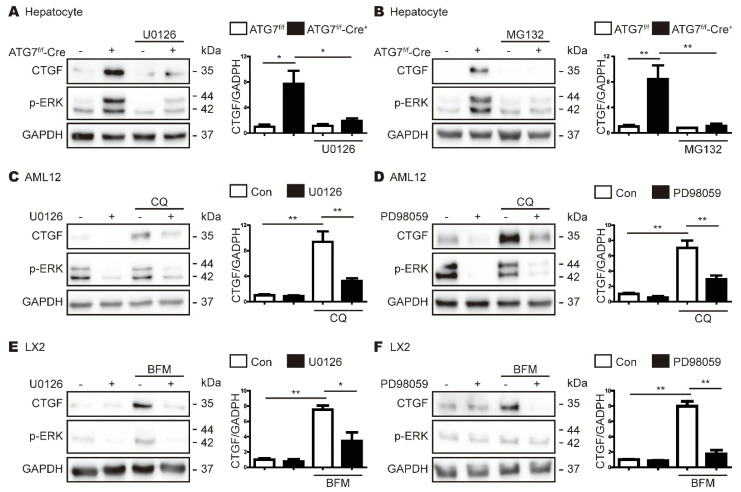
Increased CTGF expression caused by autophagy inhibition is associated with elevated p-ERK levels. (**A**,**B**) Western blot analyses of CTGF in primary hepatocytes from Cre-negative and Cre-positive ATG7^flox/flox^ (ATG7^f/f^ and ATG7^f/f^-Cre^+^) mice. The hepatocytes were treated with or without 10 μM U0126 (**A**) or 1 μM MG132 (**B**) for 24 h. The expression of tubulin was measured as a loading control. The data in the bar graphs represent the mean ± SEM of three independent measurements; * *p* < 0.05, ** *p* < 0.01. (**C**,**D**) Western blot analyses showing the effects of ERK inhibitors on the CQ-induced expression of CTGF. AML12 cells were treated with CQ and 10 μM U0126 (**C**) or 40 μM PD98059 (**D**) for 24 h. GAPDH expression was measured as a loading control. The data in the bar graph represent the mean ± SEM of three independent measurements; ** *p* < 0.01. (**E**,**F**) Western blot analyses showing the effects of ERK inhibitors on the BFM-induced expression of CTGF and p-ERK. LX2 cells were incubated with BFM and 10 μM U0126 (**E**) or 40 μM PD98059 (**F**) for 24 h. GAPDH expression was measured as a loading control. The data in the bar graph represent the mean ± SEM of three independent measurements; ** *p* < 0.01.

Next, in order to confirm the association between increased levels of CTGF and p-ERK, primary hepatocytes from ATG7^f/f^-Cre^+^ mice were treated with the ERK inhibitors PD98059 and U0126, as well as MG132, which reduces ERK phosphorylation [20]. The suppression of p-ERK expression with U0126 or MG132 reduced CTGF expression in primary hepatocytes from ATG7^f/f^-Cre^+^ mice (Figure 5A,B). Similarly, CQ- or BFM-induced CTGF expression was reduced significantly following treatment of AML12 and LX2 cells with PD98059 or U0126 (Figure 5C–E). Overall, these results suggest that the autophagy deficiency-induced increase in CTGF expression is mediated by the upregulation of ERK phosphorylation.

### 3.7. Inhibition of Autophagy Also Increases CTGF Expression in Kidney Cells

**Figure 6:**
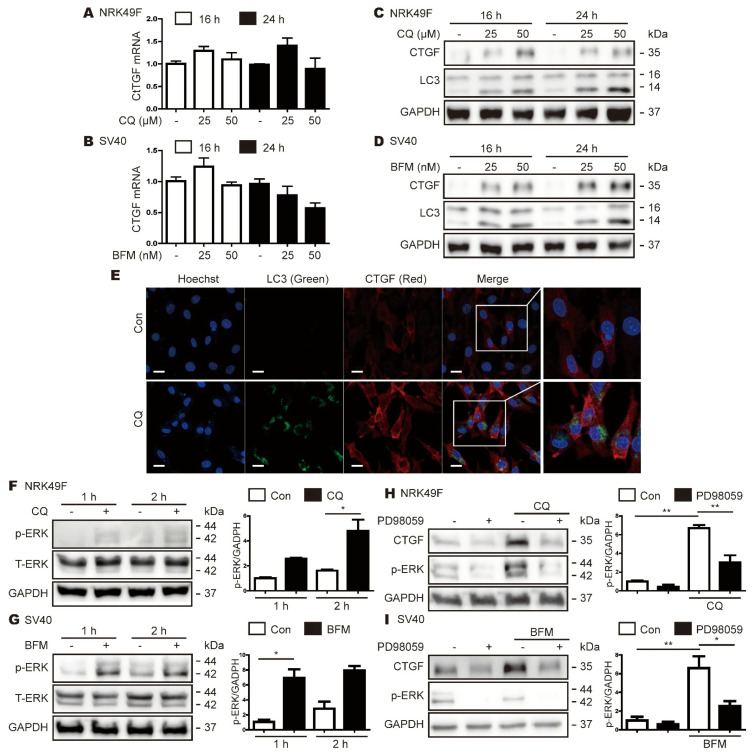
Inhibition of autophagy increased p-ERK and CTGF expression in kidney cells. NRK49F cells were treated with 25 or 50 µM CQ for 24 h, and SV40 MES 13 cells were treated with 25 or 50 nM BFM for 16 or 24 h. (**A**,**B**) Real-time RT-PCR analyses of CTGF mRNA expression in CQ-treated NRK49F cells (**A**) and BFM-treated SV40 MES 13 cells (**B**). (**C**,**D**) Western blot analyses showing the effects of CQ and BFM on CTGF and LC3 expression in NRK49F (**C**) and SV40 MES 13 (**D**) cells. (**E**) IF analyses showing the colocalization of CTGF (red) with LC3 (green) in AML12 cells in the presence or absence of CQ. (**F**,**G**) Western blot analyses showing the effects of CQ and BFM on p-ERK and total ERK (T-ERK) expression levels in NRK49F (**F**) and SV40 MES 13 (**G**) cells, respectively. The data in the bar graphs represent the mean ± SEM of three independent measurements; * *p* < 0.05. (**H**,**I**) Western blot analyses showing the effects of PD98059 (40 μM) on CQ- or BFM-induced CTGF expression. NRK49F cells were incubated with CQ for 24 h (**H**), and SV40 MES 13 cells were incubated with BFM for 24 h (**I**). The data in the bar graphs represent the mean ± SEM of three independent measurements; * *p* < 0.05, ** *p* < 0.01.

Previous studies have reported that autophagy has a significant effect on kidney function and the maintenance of homeostasis [21]; therefore, we examined whether autophagy inhibition increases CTGF expression in kidney cells. Consistent with the results seen in hepatocytes, CQ- or BFM-mediated inhibition of autophagy did not affect CTGF mRNA expression in NRK49F and SV40 MES 13 cells (Figure 6A,B), but did increase CTGF protein expression in both cell types (Figure 6C,D). CQ also increased the colocalization of LC3 and CTGF in NRK49F cells (Figure 6E). In addition, inhibition of autophagy increased p-ERK expression in NRK49F and SV40 MES 13 cells (Figure 6F,G), and the inhibition of phospho-ERK reduced CTGF expression in CQ- or BFM-treated cells (Figure 6H,I).

## 4. Discussion

**Figure 7:**
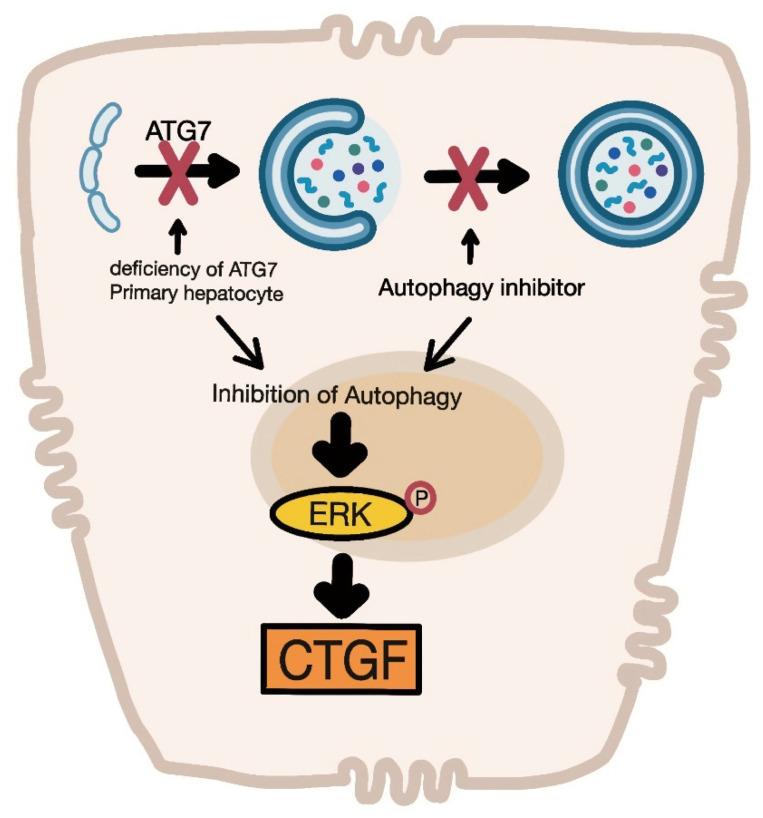
Increased levels of phosphorylated ERK induce CTGF expression in autophagy-deficient mouse hepatocytes.

In the present study, we isolated and characterized primary hepatocytes from hepatocyte-specific ATG7 knockout mice. The main observation in this study was that the expression levels of the CTGF and p-ERK proteins were elevated significantly in primary hepatocytes from autophagy-deficient mice. The inhibition of autophagy also increased the expression levels of both CTGF and p-ERK in mammalian hepatic cell lines (Figure 7).

CTGF is upregulated by TGF-β and mediates fiber and matrix interactions [5,6]. Previous studies have shown that liver-specific atg5 knockout mice display hepatic fibrosis alongside the increased transcription of TGF-β target genes such as CTGF, collagen, and α-SMA [14]. Here, we isolated primary hepatocytes from ATG7-knockout mice and examined the expression levels of fibrotic markers because the deletion of ATG7 by albumin-Cre mainly affects hepatocytes (Supplementary Figure S2). Notably, primary hepatocytes from autophagy-deficient ATG7^f/f^-Cre^+^ mice displayed increased levels of CTGF expression, but there were no consistent effects of ATG7 knockout on the activation of TGF-β. Another paper reported [15] an increase in YAP and CTGF, a target of Yap, in autophagy deficient liver. However, our results showed that both YAP mRNA and protein were reduced in primary hepatocytes from ATG7^f/f^-Cre^+^ mice (Supplementary Figure S3), and further studies are needed for these different results.

In line with the findings in primary mouse hepatocytes, in vitro analyses confirmed that the CQ- or BFM-mediated inhibition of autophagy increased CTGF protein expression in two mammalian hepatic cell lines. Ubiquitin accumulation and p62 aggregation are increased following the liver-specific inhibition of autophagy, and the increased p62 expression leads to the sustained activation of NRF2 [22]. Subsequently, activated NRF2 increases the expression of antioxidant genes and paradoxically increases liver damage [23]. However, we found that CTGF expression was still induced in AML12 cells following the small interfering RNA (*siRNA*)-mediated knockdown of SQSTM1/p62 (Supplementary Figure S4), confirming that the autophagy deficiency-mediated stabilization of CTGF protein expression does not involve SQSTM1/p62.

We also investigated the effect of the *siRNA*-mediated silencing of ATG7 on CTGF expression. The knockdown of ATG7 in AML12 cells showed a tendency to increase CTGF protein levels; however, there were no obvious changes in the levels of the LC3 II and SQSTM1/p62 proteins (Supplementary Figure S5). These findings indicate that a deficiency of ATG7 cannot inhibit autophagy completely, and that increased CTGF expression results from a general inhibition of the autophagy pathway rather than a loss of ATG7 alone. 

In a previous study, Bernard et al. [24] reported that autophagy is an upstream regulator of CTGF. In that study, CTGF expression in fibroblasts was reduced following treatment with LY294002 (a PI3 kinase inhibitor) and 3MA (an autophagy inhibitor). By contrast, in our current study, 3MA did not have any obvious effects on the levels of the LC3 and SQSTM1/p62 proteins in AML12 cells (Supplementary Figure S6). Another study reported that 3MA has a dual effect on autophagy regulation [25], as it is not a specific class III PI3 kinase inhibitor and can inhibit the activation of class I PI3 kinases, which may enhance autophagy. 

Autophagy inhibitors impair lysosomal function by inhibiting lysosomal acidification. However, we found that lysosomal acidification was comparable in primary hepatocytes from ATG7^f/f^-Cre^+^ and control mice (ATG7^f/f^) (Supplementary Figure S7). Taken together, our results indicate that CTGF expression is increased when autophagic flux is inhibited. 

We found that ERK phosphorylation was significantly higher in primary hepatocytes from ATG7^f/f^-Cre^+^ mice than in those from control mice. However, the physiological consequences of changes in ERK phosphorylation in cells with abnormal autophagy have not yet been determined. For example, Mahli et al. [26] reported that autophagy impairment induces hepatic steatosis and inflammation through ERK activation. In contrast to the results presented here, another group reported that the deletion of ATG7 or the blocked of LC3 lipidation reduced ERK phosphorylation [27]; however, in that study, ATG7 deficiency increased the total ERK1 levels in the liver homogenate fraction. Here, we found that both p-ERK and total ERK levels were increased in primary hepatocytes from ATG7^f/f^-Cre^+^ mice, and that p-ERK expression was increased by autophagy inhibitors. In addition, we confirmed that the reduction of p-ERK also reduced the autophagy deficiency-induced increase in CTGF expression.

CTGF expression is increased in renal fibrosis as well as hepatic fibrosis, and a recent study reported CTGF as a new target in renal fibrosis [28]. In addition, autophagy is essential for the maintenance of kidney homeostasis, structure, and function, and dysregulated autophagy contributes to the pathogenesis of various kidney diseases [29]. There are many reports that autophagy also modulates kidney disease [30]. Therefore, we performed experiments using cultured kidney cells. Consistent with the findings in hepatocytes, CTGF and phospho-ERK protein levels were increased upon the inhibition of autophagy in kidney cells.

In summary, the results presented here demonstrate that autophagy plays a key role in the regulation of the expression of CTGF in hepatocytes. Our findings show that autophagy inhibition is associated with increased CTGF expression and ERK phosphorylation in hepatocytes and renal cells. 

## Data Availability

Not applicable.

## Supplementary Materials

The following are available online at https://www.mdpi.com/article/10.3390/cells11172704/s1. Figure S1: Western blot analyses of phospho-STAT3 (p-STAT3), total-SMAD3 (T-SMAD3), p-ERK, and total ERK (T-ERK) in primary hepatocytes from ATG7^f/f^ and ATG7^f/f^-Cre^+^ mice after treatment with or without TGF-β (5 ng/mL) for the indicated times. The expression of GAPDH was measured as a loading control. Figure S2: Real-time RT-PCR analyses of the ATG7 mRNA level in primary hepatocytes, primary hepatic stellate cell (HSC) and hepatic kupffer cell (KC) from ATG7^f/f^ and ATG7^f/f^-Cre^+^ mice. Figure S3: Real-time RT-PCR analyses of the YAP mRNA level and Western blot analyses of YAP protein expression in primary hepatocytes from ATG7^f/f^ and ATG7^f/f^-Cre^+^ mice. Figure S4: Western blot analysis showing the effect of small interfering RNA (siRNA)-SQSTM1/p62 on CQ-stimulated CTGF protein expression. AML12 cells were transfected with 100 nM siRNA-SQSTM1/p62 or control siRNA, and then treated with or without CQ. Figure S5: Western blot analysis showing the effect of small interfering RNA (siRNA)-ATG7 on CTGF, p62 and LC3 protein expression in AML12 cells. Figure S6: Western blot analyses showing the effects of 3MA on ATG7, CTGF, p62 and LC3 expression in AML12 cells. Figure S7: Lysotracker staining was performed in primary hepatocytes from ATG7^f/f^ and ATG7^f/f^-Cre^+^ mice.

## Abbreviations

α-SMA: α-smooth muscle actin; ATG7: autophagy-related 7; BFM: bafilomycin A1; CQ: chloroquine; CTGF: connective tissue growth factor; ERK: extracellular signal-regulated kinase; FBS: fetal bovine serum; IF: immunofluorescent; PAI-1: plasminogen activator inhibitor-1; TGF-β: transforming growth factor-β
