# Supplementary material for: Increased Levels of Phosphorylated ERK Induce CTGF Expression in Autophagy-Deficient Mouse Hepatocytes
Source: Cells. 2022 Aug 30;11(17):2704. doi: 10.3390/cells11172704 (PMC9454551; doi:10.3390/cells11172704)
Supplement: Supplementary file 1 [file cells-11-02704-s001.zip › cells-1884546-supplementary.pdf]

cells MDPI

Supplementary Materials

Increased Levels of Phosphorylated ERK Induce CTGF Expression in Autophagy-Deficient Mouse Hepatocytes

Materials and Methods

3-Methyladenine (3MA, M9281) was purchased from Sigma-Aldrich. An anti-phospho-STAT3 (Tyr705) (CS9138), anti-STAT3 (CS4904), anti-phospho-JNK (CS9251), anti-JNK (CS3252), anti-phospho-p38 (CS9211), anti-p-38 (CS9212) and anti-Yap (CS4912) antibodies were purchased from Cell Signaling Technology.

Small Interfering RNA (siRNA)-Mediated Depletion of ATG7

A pre-designed siRNA targeting ATG7 (*siATG7*) (SC41448) and a scrambled control siRNA (*siCon*) (SC37007) were purchased from Santa Cruz Biotechnology. AML12 Cells were transfected with 100 nM siRNA using Lipofectamine RNAiMAX (Thermo Scientific, 13778-075) for 5 h, cultured in medium containing 0.5% FBS, and harvested ~48 h after transfection.

Lysotracker Staining

For lysotraker staining, primary hepatocytes from Cre-negative and Cre-positive Atg7$^{flox/flox}$ (Atg7$^{f/f}$ and Atg7$^{f/f}$-Cre^{+}) mice were stained with 100 nM Lysotracker Red DND-99 (Invitrogen, L7528) for 10 min at 37°C, and the hepatocytes were imaged using a microscope.

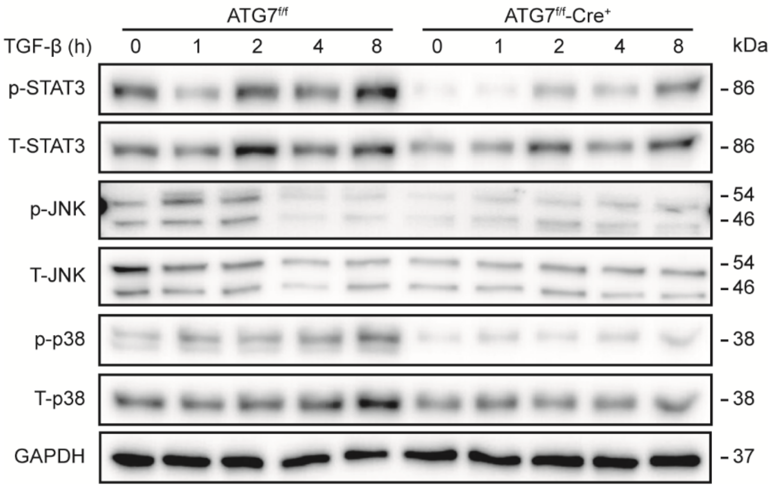

Supplementary Figure S1. Western blot analyses of phospho-STAT3 (p-STAT3), total-SMAD3 (T-SMAD3), p-ERK, and total ERK (T-ERK) in primary hepatocytes from Atg7$^{f/f}$ and Atg7$^{f/f}$-Cre^{+} mice after treatment with or without TGF-β (5ng/ml) for the indicated times. The expression of GAPDH was measured as a loading control.

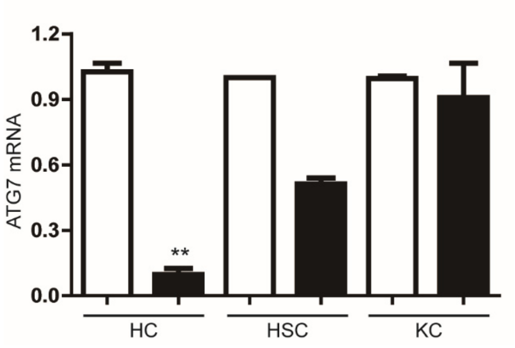

Supplementary Figure S2. Real time RT-PCR analyses of ATG7 mRNA level in primary hepatocytes, primary hepatic stellate cell (HSC) and hepatic kupffer cell (KC) from Atg7$^{f/f}$ and Atg7$^{f/f}$-Cre^{+} mice.

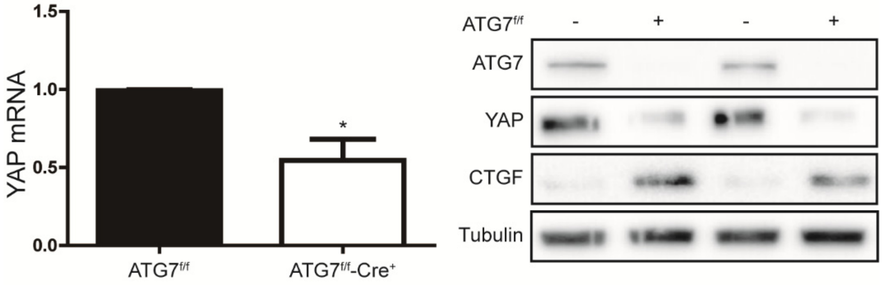

Supplementary Figure S3. Real time RT-PCR analyses of YAP mRNA level and western blot analyses of YAP protein expression in primary hepatocytes from Atg7$^{f/f}$ and Atg7$^{f/f}$-Cre^{+} mice.

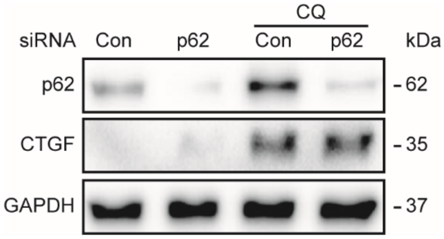

Supplementary Figure S4. Western blot analysis showing the effect of small interfering RNA (*siRNA*)-SQSTM1/p62 on CQ-stimulated CTGF protein expression. AML12 cells were transfected with 100nM *siRNA-SQSTM1/p62* or *control siRNA*, and then treated with or without CQ. .

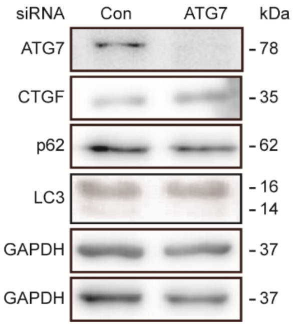

Supplementary Figure S5. Western blot analysis showing the effect of small interfering RNA (*siRNA*)-ATG7 on CTGF, p62 and LC3 protein expression in AML12 cells.

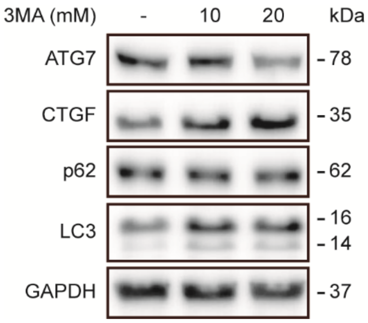

Supplementary Figure S6. Western blot analyses showing the effects of 3MA on ATG7, CTGF, p62 and LC3 expression in AML12 cells.

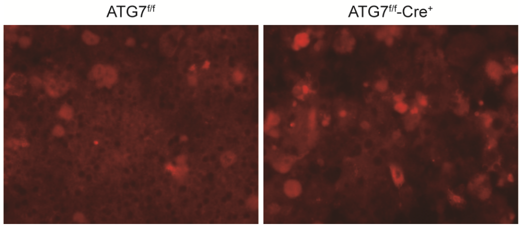

Supplementary Figure S7. Lysotracker staining was performed in primary hepatocytes from Atg7$^{f/f}$ and Atg7$^{f/f}$-Cre^{+} mice.
